# Isoquercetin and Zafirlukast Cooperatively Suppress Tumor Growth and Thromboinflammatory Signaling in a Xenograft Model of Ovarian Cancer

**DOI:** 10.1096/fj.202502774R

**Published:** 2025-12-31

**Authors:** Justine A. Keovilay, Jason W. Hoskins, Jean‐Pierre Kinet, Thomas C. Lines, Daniel R. Kennedy

**Affiliations:** ^1^ College of Pharmacy and Health Sciences Western New England University Springfield Massachusetts USA; ^2^ Institute for Cardiovascular & Metabolic Research, School of Biological Sciences University of Reading Reading UK; ^3^ Laboratory of Translational Genomics National Cancer Institute, National Institutes of Health Bethesda Maryland USA; ^4^ Department of pathology at Beth Israel Deaconess Medical Center Boston Massachusetts USA; ^5^ Quercis Pharma AG Zug Switzerland; ^6^ Department of Medicine UMass Chan Medical School‐Baystate Springfield Massachusetts USA

## Abstract

Cancer‐associated thrombosis (CAT), encompassing both venous thromboembolism and arterial thrombosis, contributes to up to 14% of cancer‐related mortality and remains difficult to treat due to the bleeding risks of conventional anticoagulants. Protein disulfide isomerase (PDI) and its family member ERp57 (PDIA3) are thiol isomerases that regulate both arterial and venous thrombosis and are also upregulated in tumors, where they promote growth, metastasis, and immune evasion. Here, we evaluated the therapeutic potential of two thiol isomerase inhibitors—isoquercetin (ISOQ), a selective PDI inhibitor, and zafirlukast (ZAF), a broad‐spectrum inhibitor of thiol isomerases such as PDI and ERp57—individually and in combination, in a xenograft model of ovarian cancer. ISOQ inhibited both platelet aggregation and Factor Xa generation induced by tumor cells and significantly suppressed tumor growth, thromboinflammatory markers, and expression of tissue factor, VEGF, TMEM176B, and PD‐L1. ISOQ also potentiated standard cisplatin/gemcitabine chemotherapy. Notably, the combination of low‐dose ISOQ plus ZAF achieved ≥ 80% inhibition of key tumor‐associated markers at one‐third the monotherapy dose and outperformed either agent alone. These findings support ISOQ and ZAF as promising agents for the treatment of cancer and CAT and establish thiol isomerase inhibition as a strategy to simultaneously target thrombosis, tumor progression, and immune escape.

## Graphical abstract

Treatment using either the thiol isomerase inhibitor isoquercetin or zafirlukast lowers tissue factor levels, decreases VEGF, inhibits PD‐L1, and TMEM176B in a xenograft model of ovarian cancer. A combination of the two agents, at threefold lower concentrations than either drug, significantly enhanced the inhibition further, suggesting a dual action of the drug in combination may be a promising treatment for cancer and cancer‐induced thrombosis.

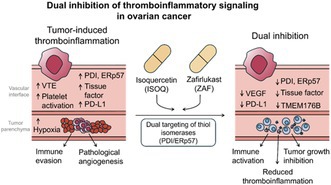

## 1 Introduction

Hemostatic complications are a common and clinically significant phenomenon in patients with solid tumors. Overall annual death rates are almost threefold higher for cancer patients with arterial thrombosis, and 50‐fold higher for cancer patients with venous thromboembolism (VTE), compared to the general population [1], which is the second leading cause of cancer‐related mortality after disease progression itself [2]. Beyond its clinical consequences, thrombosis actively contributes to tumor progression as cancer cells exploit the coagulation system to create a fibrin‐rich microenvironment that impairs perfusion, drives hypoxia, and fosters immune evasion and pathological angiogenesis [3]. In this context, the thrombotic reaction is not merely a complication but a functional enabler of cancer growth and metastasis. While anticoagulants remain standard for VTE treatment, mounting evidence suggests they may also interfere with tumor biology [4], underscoring the therapeutic potential of targeting the thromboinflammatory axis in cancer.

While the relationship between cancer and increased thrombotic activity is complex and multifactorial, increased risk of cancer‐induced thrombosis is correlated with increased levels of protein disulfide isomerase (PDI), a key thiol isomerase [5]. PDI and its related thiol isomerase family members, ERp57, ERp5, and ERp72, play a unique and crucial role in the coagulation cascade [6, 7, 8, 9, 10]. PDI is secreted by activated platelets and endothelial cells and is critical for thrombus formation [11]. Uniquely, targeting thiol isomerases offers a means to blunt both arterial (platelet‐rich) and venous (fibrin‐rich) thrombosis without disrupting normal hemostasis, as inhibiting their activity in vivo blocks pathological thrombus formation yet does not provoke bleeding [11, 12, 13, 14, 15].

Thiol isomerases are also upregulated in many distinct cancer types [16, 17, 18, 19], and increased levels of thiol isomerases have been positively correlated to increased oncogenic transformation [20], gene transcription [21], and metastasis [22], as well as implicated in shaping the tumor immune microenvironment, including modulation of antigen presentation and immune checkpoint signaling such as PD‐L1 expression [23]. Therefore, thiol isomerase inhibitors hold significant promise as dual‐acting agents, offering a novel approach to address the challenges of both thrombosis and cancer progression. However, despite this potential, inhibitors of thiol isomerases are primarily explored for either their antithrombotic (rutin [14], galangin [24], and piericones [25]) or anticancer (PACMA‐31 [26], CCF642 [27], and aziridine‐2‐carboxlic acid derivatives [28]) effects.

The notable exception is zafirlukast (ZAF), an FDA‐approved medication that we have identified through a high‐throughput screen, which inhibited the thiol isomerase ERp57 [29]. Further exploration demonstrated broad thiol isomerase inhibition, affecting PDI, ERp5, and ERp72, unlike most other inhibitors that are PDI selective [29]. ZAF demonstrated inhibition of platelet aggregation, fibrin formation, P‐selectin expression, and intravital thrombus formation [29]. ZAF also inhibits the growth of numerous cancer cell lines, inhibits tumor growth in xenografted mice, provides additive effects in combination with cisplatin/gemcitabine treatment in xenografted mice, and inhibits metastasis in mice [29]. In a small clinical trial, ZAF delayed the rate of rise of a key tumor marker (CA‐125) in four ovarian cancer patients at risk of tumor marker‐only relapse [29]. While CA‐125 reduction in ovarian cancer patients at risk of tumor marker‐only relapse was a promising result, further enhancing ZAF's efficacy was considered to be valuable. Chemical optimization of zafirlukast using structure–activity‐relationship to improve binding to thiol isomerases, while also eliminating the moiety of the compound responsible for leukotriene receptor antagonism, was explored using two iterative optimizations totaling 35 analogs [30]. The optimized analogs maintained or slightly improved antithrombotic [30] and anticancer activity [29] compared to zafirlukast, demonstrating the leukotriene receptor was not a significant contributor to antithrombotic or anticancer activity but did not lead to a notable increase in efficacy [29, 30].

Thus, we determined that the best way to potentially improve zafirlukast's efficacy would be in combination with another pharmacologic agent. In this work, we chose to explore isoquercetin (ISOQ), a naturally occurring flavonoid (a glucoside of quercetin) that selectively inhibits PDI [31]. In a phase II trial of 64 high‐risk patients with cancer, ISOQ treatment increased plasma PDI inhibitory activity and significantly decreased markers of hypercoagulability, with no VTE events or major hemorrhages observed [31]. Less is known about ISOQ ‘s direct anti‐cancer properties; however, one short‐term study found decreased colon cancer growth and vascularization in mice [32].

Although developed for different clinical indications, ISOQ and ZAF converge on a shared thiol isomerase–dependent mechanism that underlies both thrombosis and malignancy. Thus, we hypothesized that combining these agents could achieve complementary inhibition of PDI and ERp57, yielding enhanced antithrombotic and antitumor effects. The aim of this study was to first evaluate the therapeutic and mechanistic effects of ISOQ on tumor progression, CAT, and immune modulation in a xenograft model of ovarian cancer. Then, to determine the effects of adding ISOQ in combination with ZAF on those processes. Herein, we demonstrate that dual thiol isomerase inhibition with ISOQ plus ZAF markedly attenuates ovarian cancer progression and prothrombotic tendency while favorably altering the tumor immune microenvironment, compared with either agent alone.

## 2 Methods

In our xenograft model, the study exclusively examined female mice because ovarian cancer is only relevant in females.

### 2.1 Reagents

ISOQ was provided by Quercis Pharma (Zug, Switzerland). ZAF was purchased from TCI America (Portland, OR, USA). Dieosinediglutathione (DiEGSSG), cisplatin, and gemcitabine were purchased from Cayman Chemical (Ann Arbor, Michigan, USA).

Recombinant human PDI, angiogenesis and inflammation array kits, as well as antibodies against TMEM176B (LR8), PDI, and ERp57, were purchased from Abcam (Waltham, MA, USA). Antibodies against tissue factor, VEGF, PD‐L1, β‐actin, along with secondary antibodies and blue loading buffer, were obtained from Cell Signaling Technology (Danvers, MA, USA).

Recombinant coagulation factors VIIa (FVIIa) and X (FX), and the fluorescent Factor Xa cleaving substrate were purchased from Prolytix (Essex Junction, VT, USA).

Protein quantification and lysis reagents, including Pierce BCA (Bradford) Plus and detergent‐compatible assays, mPER mammalian protein extraction reagent, and Halt protease and phosphatase inhibitor cocktail, were purchased from Thermo Fisher Scientific (Waltham, MA, USA), as were RNAlater and P‐selectin ELISA kits.

Recombinant bovine insulin, dithiothreitol (DTT), bovine serum albumin (BSA), standard laboratory buffers, and other chemicals were purchased from Sigma‐Aldrich (St. Louis, MO, USA) and VWR (Radnor, PA, USA). Clear‐bottom 96‐well and 384‐well plates were obtained from Corning (Corning, NY, USA).

### 2.2 Cell Culture

Human ovarian adenocarcinoma (OVCAR8) (NCI‐DTP Cat#OVCAR‐8, RRID: CVCL_1629) was cultured in RPMI 1640 medium supplemented with 10% fetal bovine serum (FBS), 1% (*w*/*v*) sodium pyruvate, 10 mM HEPES, 2 mM glutamine, and 100 IU/mL penicillin/streptomycin.

### 2.3 Thiol Isomerase Activity Assay

OVCAR8 cells were seeded in black 96‐well plates at a density of 10 000 cells per 100 μL of complete medium. The following day, wells were washed with 100 μL of potassium phosphate buffer (100 mM potassium phosphate (pH 7.4), containing 2 mM EDTA) and then replenished with 80 μL of the same buffer. Experimental wells were treated with ISOQ at concentrations of 1, 3, 10, or 30 μM for 10 min at room temperature. Immediately after incubation, DTT was added to a final concentration of 5 μM, followed by DiEGSSG fluorescent probe at 150 nM. Fluorescence was measured kinetically (excitation: 520 nm; emission: 550 nm) for 30 min using a BioTek Synergy H1 microplate reader (Agilent, Santa Clara, CA, USA). For mouse plasma samples, 70 μL of plasma was added to each well along with 10 μL of potassium phosphate buffer. Final concentrations of 5 μM DTT and 150 nM DiEGSSG were added immediately before measurement under the same fluorescence settings. Data were normalized to the untreated control for each condition, with raw values reported as relative fluorescent units per minute (RFU/min).

### 2.4 Factor Xa Generation

OVCAR8 cells were seeded at a density of 100 000 cells per well in clear 12‐well plates and incubated overnight. The following day, cells were then washed with 1 mL of Tris‐buffered saline (TBS; 15 mM Tris–HCL, 4.6 mM Tris‐base, 150 mM NaCl, pH 7.6, adjusted at room temperature), and incubated with ISOQ at concentrations of 1, 3, 10, or 30 μM in 500 μL of TBS for 30 min at room temperature.

After ISOQ treatment, an additional 500 μL of TBS was added to each well along with the following components: 1 μL of fluorescent substrate, 5 nmol/L Factor VIIa (FVIIa), 150 nmol/L of Factor X (FX), and 5 mmol/L of CaCl_2_. Fluorescence was measured kinetically (excitation: 352 nm; emission: 470 nm) for 45 min using a SpectraMax M3 microplate reader (Molecular Devices, Sunnyvale, CA, USA).

### 2.5 Platelet Preparation

Following approval from the Western New England University Institutional Review Board (IRB), human platelets were collected from consenting, drug‐free donors using standard venipuncture techniques. Blood was drawn into tubes containing 3.2% sodium citrate.

Platelet‐rich plasma (PRP) was prepared by centrifuging whole blood at 100 × *g*. Platelets were then washed using an ADP‐sensitive method: samples were centrifuged at 350 × *g* for 20 min, and the supernatant was carefully aspirated, and the platelet pellet was resuspended in Tyrode's buffer to a final concentration of 1 × 10^8^ platelets/mL. The Tyrode's buffer consisted of 20 mM HEPES, 144 mM NaCl, 3 mM KCl, 12 mM NaHCO_3_, and 1 mM MgCl_2_, pH 7.3 adjusted at room temperature.

### 2.6 Tumor Cell‐Induced Platelet Aggregation

Washed platelets were incubated with ISOQ at concentrations of 10 and 30 μM for 5 min at 37°C prior to analysis by light transmission aggregometry. Tumor cell‐induced platelet aggregation (TCIPA) was then initiated by co‐incubating platelets with 5 × 10^5^ OVCAR8 cells per sample. A negative control containing platelets without any agonist was included to confirm the absence of spontaneous aggregation following preparation.

### 2.7 Animal Studies

Immunodeficient NCG mice (Charles River Laboratories) were used to establish xenograft models using OVCAR8 cells. A total of 250 000 OVCAR8 cells suspended in 100 μL of serum‐free media were injected subcutaneously into the right flank of 4‐week‐old female mice. Tumor volume was measured twice weekly and calculated using the formula: (length×width×width)/2. Once tumors reached an average volume of 30 mm^3^, treatment was initiated. NCG mice received daily oral gavage for 46 days with one of the following: 10 mg/kg ISOQ, 30 mg/kg ISOQ, 3 mg/kg ISOQ combined with 10 mg/kg ZAF, 10 mg/kg ISOQ combined with 10 mg/kg ZAF, or vehicle control (corn oil). NOG mice were treated with one of the following regimens: chemotherapy (5 mg/kg cisplatin and 120 mg/kg gemcitabine), ISOQ alone (3 mg/kg or 30 mg/kg), or ISOQ (3 mg/kg or 30 mg/kg) in combination with cisplatin and gemcitabine. At the study endpoint, mice were euthanized by CO_2_ inhalation followed by cervical dislocation. Blood was collected via cardiac puncture for downstream analysis. Tumors were excised, fixed in 10% formalin for 24 h, and subsequently transferred to 70% ethanol for histological processing.

For the combination experiments of ISOQ and cisplatin/gemcitabine, NOG mice (Taconic Biosciences) were used to establish xenograft models using OVCAR8 cells for a better comparison to previous work with ZAF. Otherwise, the protocol remained the same as described above for the NCG mice.

### 2.8 P‐Selectin ELISA

Plasma was isolated by centrifugation of whole blood collected from mice in the xenograft study. Samples were frozen and stored at −20°C. P‐selectin levels were measured using an ELISA kit according to the manufacturer's instructions. Absorbance was measured at 450 nm using a SpectraMax M3 microplate reader (Molecular Devices, Sunnyvale, CA, USA).

### 2.9 Immunohistochemistry

Excised tumors were sectioned and stained with primary antibodies targeting tissue‐factor, VEGF, TMEM176B, PD‐L1, PDI, or ERp57, followed by incubation with an Alexa Fluor 488‐conjugated secondary antibody. Slides were imaged at 20X magnification using a Nikon Eclipse fluorescence microscope (Nikon, Tokyo, Japan), and images were captured using NIS‐Elements software (Nikon, Tokyo, Japan). Immunofluorescence signal analysis was performed using ImageJ software (NIH, Bethesda, Maryland, USA).

### 2.10 Angiogenesis and Inflammation Arrays

Tumor lysates were prepared from pooled tumors of mice treated with 10 mg/kg ISOQ, 30 mg/kg ISOQ, or vehicle control, using the lysis buffer provided in the array kit. Total protein concentrations were determined using the Pierce Detergent Compatible Bradford Assay Reagent (Thermo Fisher Scientific, Waltham, MA, USA). Array membranes were blocked at room temperature for 30 min with the kit‐supplied blocking buffer. A total of 1000 μg of protein from each treatment group was incubated with the corresponding array membrane overnight at 4°C. The membranes were then washed with the provided wash buffer and incubated with a biotinylated antibody cocktail for 2 h at room temperature. After a second wash, membranes were incubated with horse radish peroxidase (HRP)‐conjugated streptavidin for an additional 2 h at room temperature, followed by a final wash. Detection was performed using the kit‐supplied chemiluminescent detection buffer for 2 min at room temperature. Membranes were imaged using a Bio‐Rad ChemiDoc imaging system (Bio‐Rad, Hercules, CA, USA).

### 2.11 Statistics

Statistical analyses were performed using GraphPad Prism (Version 9.4.0, San Diego, CA). Data are presented as the mean ± standard deviation (SD). Depending on the experimental design, statistical significance was evaluated using an unpaired *t*‐test, a one‐way ANOVA with Dunnett's post hoc test, or a two‐way ANOVA with Tukey's or Sidak's post hoc test. Statistical significance was defined as follows: *p < 0.05, **p < 0.01, *** *p* < 0.001, or **** *p* < 0.0001.

### 2.12 Study Approval

All animal procedures were conducted in accordance with institutional guidelines and were approved by the IACUC and Institutional Biosafety Committee (IBC) of UMass Chan Medical School‐Baystate.

## 3 Results

### 3.1 Isoquercetin Inhibits Thiol Isomerase Activity and Prothrombotic Activity In Vitro

**FIGURE 1:**
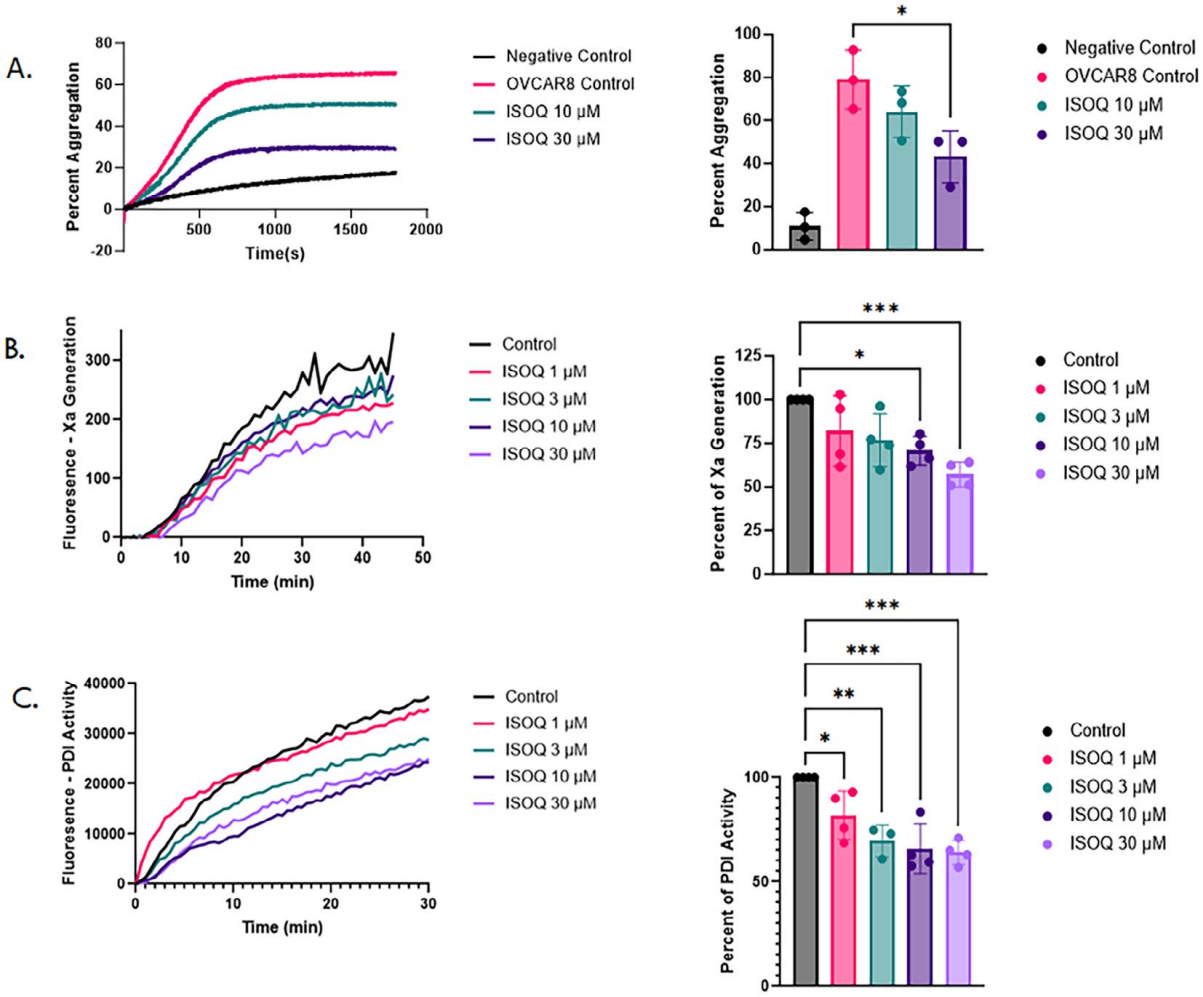
Isoquercetin inhibits key drivers of cancer‐associated thrombosis in vitro. (A) ISOQ suppresses ovarian cancer (OVCAR8) tumor cell‐induced platelet aggregation (TCIPA) (*n* = 3), where the bar graph represents endpoint aggregation. (B) ISOQ suppresses tumor cell (OVCAR8) generated Factor Xa generation (*n* = 4), where the bar graph represents inhibition at 45 min. (C) ISOQ suppresses tumor cell (OVCAR8) PDI activity (*n* = 4), where the bar graph represents inhibition at 30 min. Data are presented as mean ± SD. Statistical analysis was performed (A, B, and C) using one‐way ANOVA with Dunnett's multiple comparisons test, where *p < 0.05, **p < 0.01, and ***p < 0.001.

To assess ISOQ's antithrombotic effects, we first examined its impact on tumor cell‐induced platelet aggregation (TCPIA), a key feature of arterial thrombosis. Co‐incubation of OVCAR8 ovarian cancer cells with human platelets led to aggregation, which was reduced by 22.6% (*p* = 0.3169) at ISOQ concentrations of 10 μM and by 55.5% (*p* = 0.0123) at 30 μM (Figure 1A). We next examined tumor cell‐initiated coagulation by measuring Factor Xa generation, a functional marker of venous (coagulation‐driven) thrombosis. ISOQ treatment of OVCAR8 cells significantly reduced Factor Xa generation by 29.3% (*p* = 0.0136) at 10 μM and 42.8% (*p* = 0.0006) at 30 μM (Figure 1B). To determine whether these effects were associated with tumor cell surface thiol isomerase activity, we measured thiol isomerase enzymatic activity in OVCAR8 cells. ISOQ significantly inhibited thiol isomerase activity in a dose‐dependent manner, with reductions of 18.2% (*p* = 0.0327) at 1 μM, 30.4% (*p* = 0.0014) at 3 μM, 34.2% (*p* = 0.0002) at 10 μM, and 36% (*p* = 0.0001) at 30 μM (Figure 1C).

### 3.2 Isoquercetin Inhibits Tumor Growth and Systemic Thromboinflammatory Markers In Vivo

**FIGURE 2:**
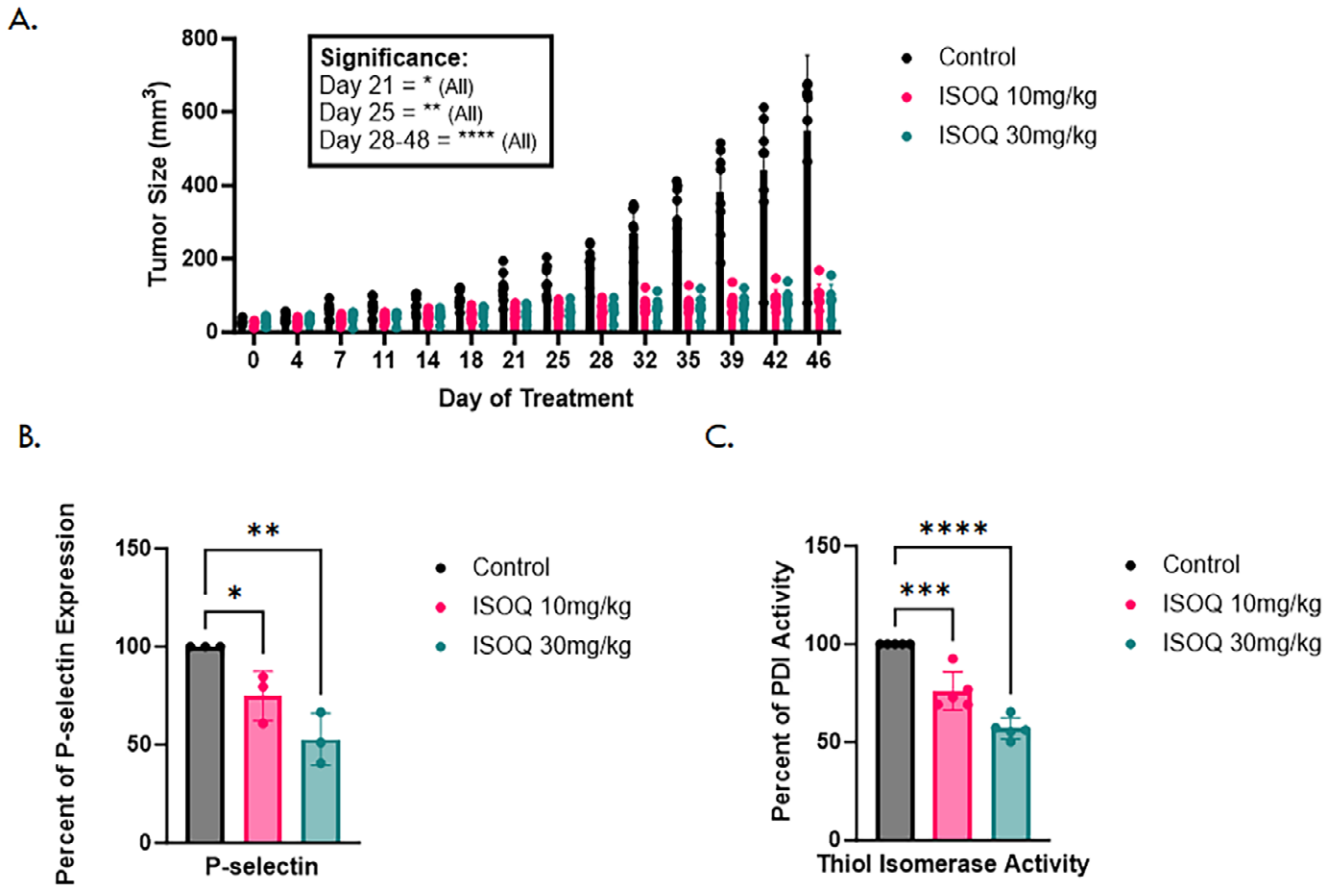
Isoquercetin suppresses ovarian tumor growth and systemic thromboinflammatory markers in vivo. (A) NCG mice were administered ISOQ via daily oral gavage, and measurements of xenografted OVCAR8 tumors were taken twice weekly. 10 mg/kg (*n* = 8) and 30 mg/kg (*n* = 8) doses demonstrated significant inhibition compared to the control (*n* = 8) by treatment day 21. (B) P‐selectin expression from pooled mouse plasma collected upon termination of the experiment (*n* = 3). (C) Thiol isomerase activity from pooled mouse plasma collected upon termination of the experiment (*n* = 4). Data are presented as mean ± SD. Statistical analysis was performed using two‐way ANOVA with Tukey's multiple comparisons test (A), or one‐way ANOVA with Dunnett's multiple comparisons test (B and C), where *p < 0.05, **p < 0.01, ***p < 0.001, and ****p < 0.0001.

To evaluate the antitumor activity of ISOQ in vivo, NCG mice bearing OVCAR8 xenograft tumors were treated via oral gavage with either 10 or 30 mg/kg ISOQ or vehicle control for 46 days. Tumor volume was monitored twice weekly. ISOQ concentrations significantly reduced tumor size, with volumetric reductions compared to control of 52.5% (*p* = 0.0283) at 10 mg/kg and 56.2% (*p* = 0.0203) at 30 mg/kg by day 21 (Figure 2A). By study completion, the final tumor burden relative to control was reduced by 81.6% (*p* < 0.0001) and 82.5% (p < 0.0001) in the 10 and 30 mg/kg groups, respectively (Figure 2A).

Next, we analyzed plasma from the xenograft‐bearing mice to evaluate circulating thromboinflammatory markers. ISOQ reduced P‐selectin, a marker of platelet activation and vascular inflammation, by 25% (*p* = 0.0465) at 10 mg/kg and 47.1% (*p* = 0.0027) at 30 mg/kg, indicating a clear dose‐dependent effect (Figure 2B). ISOQ also significantly inhibited plasma thiol isomerase activity by 23.9% (*p* = 0.0001) and 43.1% (*p* < 0.0001) at 10 and 30 mg/kg, respectively (Figure 2C).

### 3.3 Isoquercetin Suppresses Tumor‐Associated Procoagulant, Angiogenic, and Inflammatory Signaling

**FIGURE 3:**
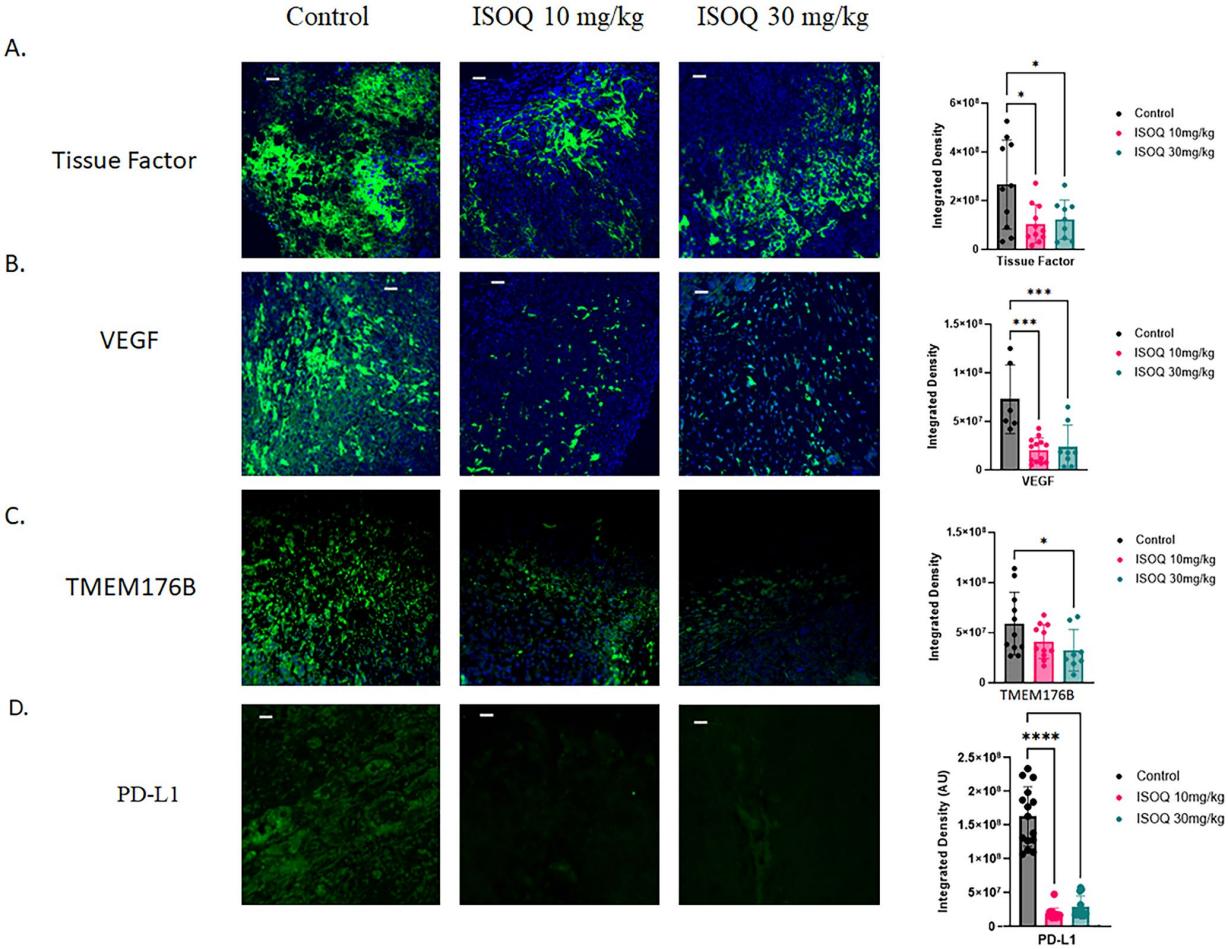
Isoquercetin suppresses procoagulant, angiogenic, and inflammation‐linked markers in ovarian tumors. Immunofluorescence analysis was performed on the excised tumors from OVCAR8 xenograft NCG mice to examine (A) tissue factor, (B) VEGF, (C) TMEM176B, and (D) PD‐L1 levels of expression. Images are representative of four to five mice per group, with three images taken per mouse of the areas of highest expression. Scale bars are 100 μm. Data are presented as mean ± SD. Data was assessed for normality and statistical analysis was performed using one‐way ANOVA (A, B, and C) with Dunnett's multiple comparisons test, where *p < 0.05 and ***p < 0.001.

To further dissect the antitumor mechanisms of ISOQ, we analyzed key molecular mediators in excised OVCAR8 tumors from the xenograft study (Figure 3). Building on our observation that ISOQ suppresses Factor Xa generation in vitro (Figure 1B), we assessed tumor‐associated tissue factor expression in vivo. Immunofluorescence staining revealed that ISOQ significantly reduced tissue factor levels by approximately 55% in both treatment groups (10 mg/kg: *p* = 0.0112; 30 mg/kg: *p* = 0.0324), consistent with its role in modulating CAT (Figure 3A). We next evaluated VEGF expression in the excised tumors, as angiogenesis plays a central role in tumor progression and metastasis [33], and chronic inflammation is known to sustain the angiogenic switch in tumors [34]. Tumors derived from ISOQ‐treated mice downregulated VEGF expression by ~70% at both 10 mg/kg (*p* = 0.0001) and 30 mg/kg (*p* = 0.0007) doses (Figure 3B). We also assessed TMEM176B, a transmembrane protein implicated in both inflammasome inhibition and immune evasion [35], and observed a 45% reduction at 30 mg/kg (*p* = 0.0461), with a similar but nonsignificant trend at 10 mg/kg (30% reduction; *p* = 0.1624) (Figure 3C).

Given ISOQ's suppressive effects on inflammatory mediators and TMEM176B, a regulator of immune escape, we also evaluated the tumors for expression of PD‐L1, a key immune checkpoint molecule [36]. ISOQ treatment significantly reduced PD‐L1 levels by over 80% at both 10 mg/kg and 30 mg/kg (*p* < 0.0001) (Figure 3D). These data suggest that ISOQ may impact inflammation‐associated immunomodulatory pathways relevant to tumor escape mechanisms.

**FIGURE 4:**
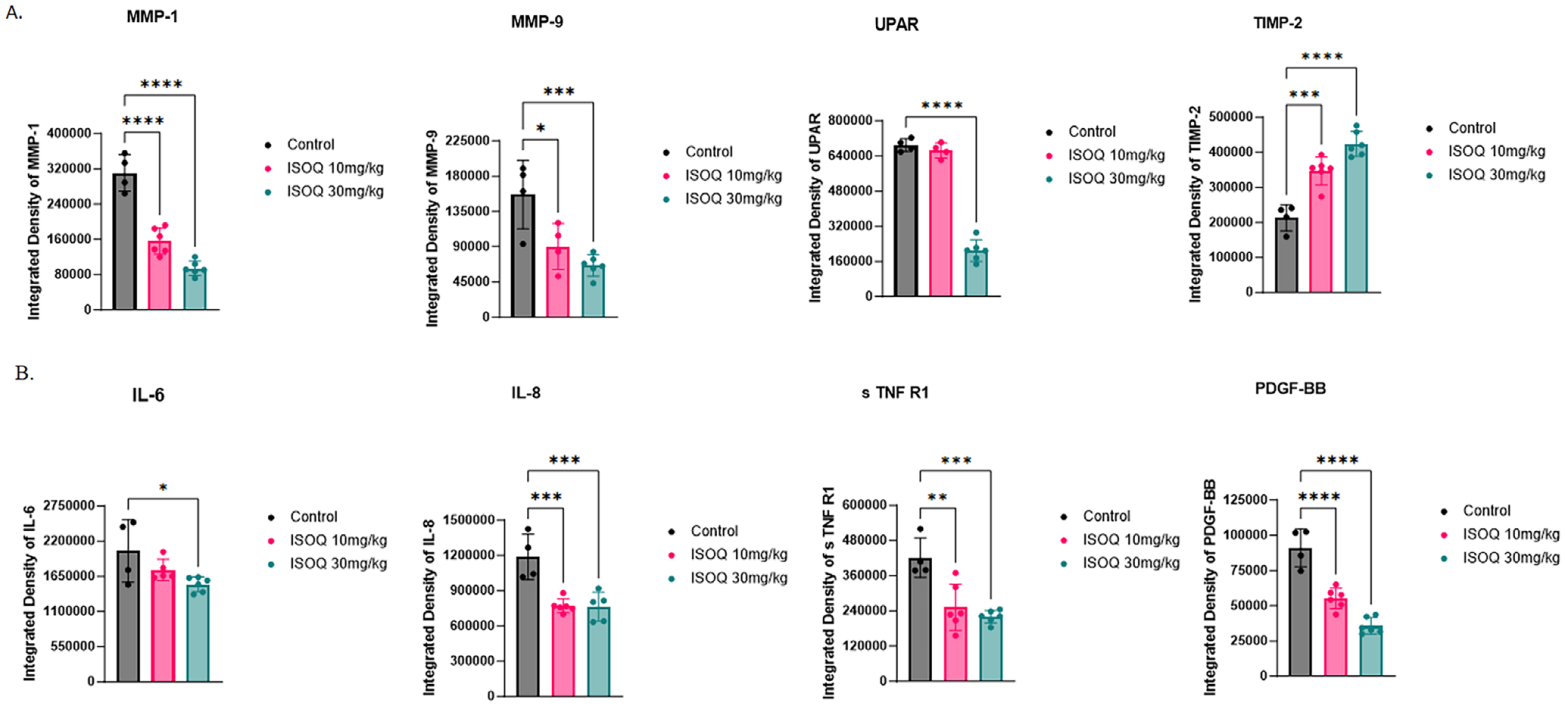
Isoquercetin modulates key angiogenesis drivers and tumor‐linked inflammation. (A) Angiogenesis and (B) inflammation arrays were performed on pooled tumor lysates (*n* = 4–6) from OVCAR8 xenograft NCG mice to examine 43 different proteins involved in these processes. Of the 43, eight proteins demonstrated significant changes in expression, including matrix metalloproteinase (MMP) 1 and 9, urokinase‐type plasminogen activator receptor (uPAR), tissue inhibitor of metalloproteinases 2 (TIMP‐2), Interleukin (IL)‐6 and IL‐8, soluble tumor necrosis factor receptor 1 (sTNF R1), and platelet‐derived growth factor BB (PDGF‐BB). Data are presented as mean ± SD. Statistical analysis was performed using one‐way ANOVA with Dunnett's multiple comparisons test, where *p < 0.05, **p < 0.01, ***p < 0.001, and ****p < 0.0001.

We extended this analysis by profiling tumor lysates derived from the xenograft study in Figure 2 using a multiplex angiogenesis array. ISOQ treatment significantly inhibited several key drivers of extracellular matrix (ECM) remodeling and vascular invasion (Figure 4A). Expression of matrix metalloproteinase‐1 (MMP‐1) was reduced by 50% with 10 mg/kg ISOQ (p = < 0.0001) and by 70% with 30 mg/kg ISOQ (*p* < 0.0001). Matrix metalloproteinase‐9 (MMP‐9) was reduced by 42% (*p* = 0.0144) and 58% (*p* = 0.001) at 10 mg/kg and 30 mg/kg, respectively. Urokinase‐type plasminogen activator receptor (uPAR) expression also declined significantly, with a 70% reduction observed at 30 mg/kg ISOQ (*p* < 0.0001). Conversely, the expression of tissue inhibitor of metalloproteinases‐2 (TIMP‐2) was markedly increased, rising by 63% at 10 mg/kg (*p* = 0.0002) and by 99% at 30 mg/kg ISOQ (*p* < 0.0001). Taken together, these findings are consistent with an overall reduction in tumor growth and angiogenesis, further supporting ISOQ's anti‐angiogenic effects and its ability to remodel the tumor ECM toward a less invasive phenotype (Figure 4A).

To further characterize ISOQ's effect on the tumor thromboinflammatory milieu, we analyzed the same tumor lysates from the xenograft study using a dedicated inflammation array. ISOQ significantly suppressed several pro‐inflammatory and pro‐tumorigenic mediators (Figure 4B). Interleukin‐8 (IL‐8) levels were reduced by 35% at 10 mg/kg (*p* = 0.0005) and 36% at 30 mg/kg (*p* = 0.0006). Soluble TNF receptor I (sTNF‐RI) expression decreased by 40% (*p* = 0.0014) and 48% (*p* = 0.0003), respectively. Platelet‐derived growth factor‐BB (PDGF‐BB) levels fell by 39% at 10 mg/kg and 61% at 30 mg/kg (both *p* < 0.0001). Finally, IL‐6 expression was reduced by 26% at 30 mg/kg ISOQ (*p* = 0.0207). These data support a broad anti‐inflammatory effect of ISOQ in the tumor microenvironment, complementing its antiangiogenic and antithrombotic activities.

### 3.4 Zafirlukast Monotherapy Inhibits Tumor Growth and Oncogenic Markers

**FIGURE 5:**
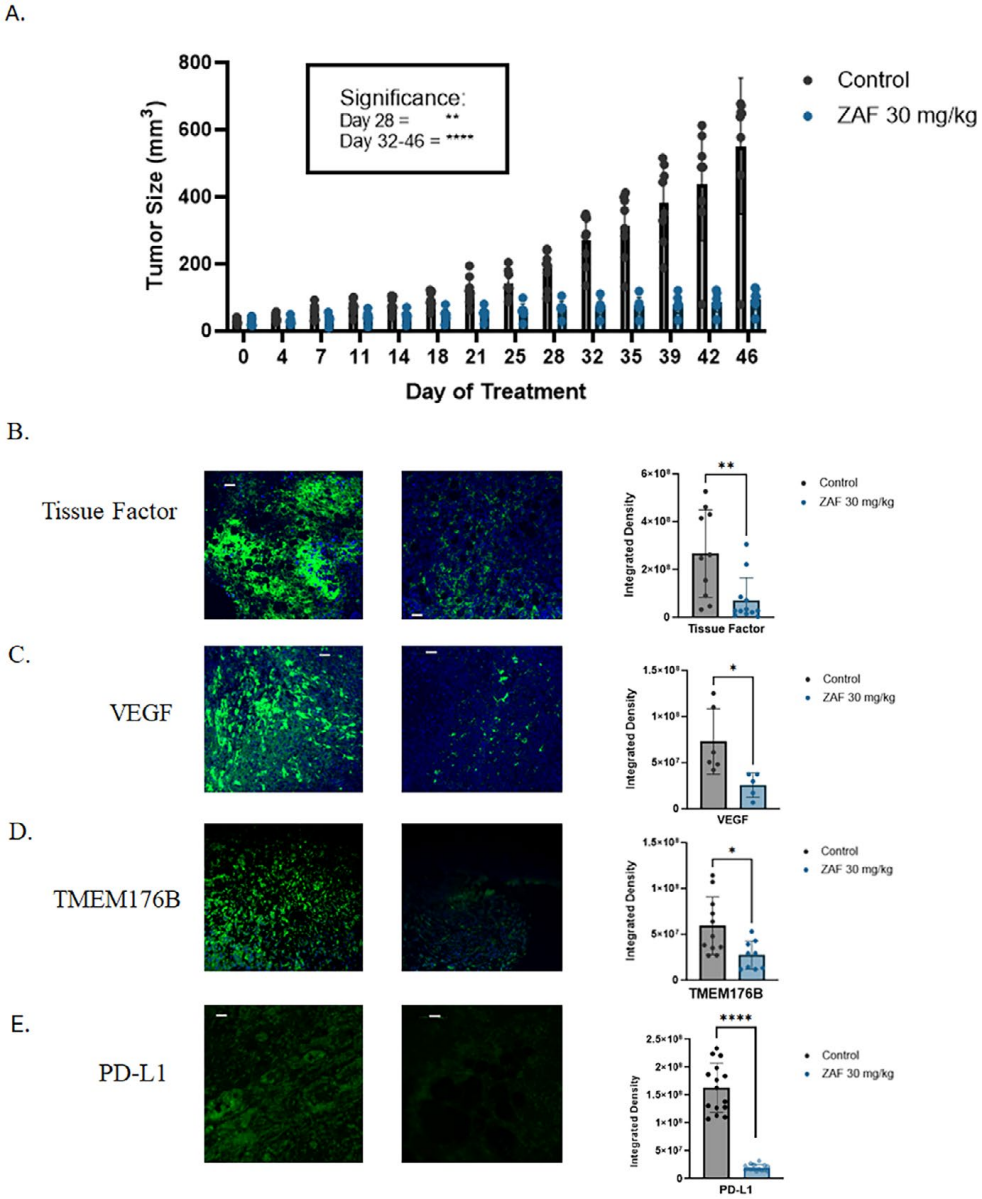
Zafirlukast monotherapy reduces tumor growth and suppresses key pro‐tumorigenic proteins in vivo. (A) NCG mice were administered ZAF via daily oral gavage, and measurements of xenografted OVCAR8 tumors were taken twice weekly. Both 10 mg/kg (*n* = 8) and 30 mg/kg (*n* = 8) doses showed significant inhibition by treatment day 25. Data are presented as mean ± SD. Excised tumors were examined for (B) tissue factor, (C) VEGF, (D) TMEM176B, and (E) PD‐L1 levels of expression. Images are representative of three to five mice per group, with three images taken per mouse of the areas of highest expression. Scale bars are 100 μm. Data are presented as mean ± SD. Statistical analysis was performed (B, C, and D) using an unpaired *t*‐test, where *p < 0.05 and **p < 0.01. Data (A) were analyzed using two‐way ANOVA with Sidak's multiple comparisons test, where **p < 0.01, and ****p < 0.0001.

As previously reported [29], ZAF monotherapy suppresses OVCAR8 tumor growth in xenograft models. To provide comparative context for combination studies, we evaluated ZAF (30 mg/kg) alone in our NCG xenograft model and confirmed a significant reduction in tumor volume by day 25 (*p* < 0.0001; Figure 5A). Immunofluorescence analysis showed moderate inhibition of tumor‐associated tissue factor, VEGF, and TMEM176B expression and strong inhibition of PD‐L1 compared to control (Figure 5 B‐E). These findings are consistent with prior results and reinforce ZAF's role as a thiol isomerase–targeting agent with efficacy in this model.

### 3.5 Complementary Thiol Isomerase Selectivity of Isoquercetin and Zafirlukast In Vivo

**FIGURE 6:**
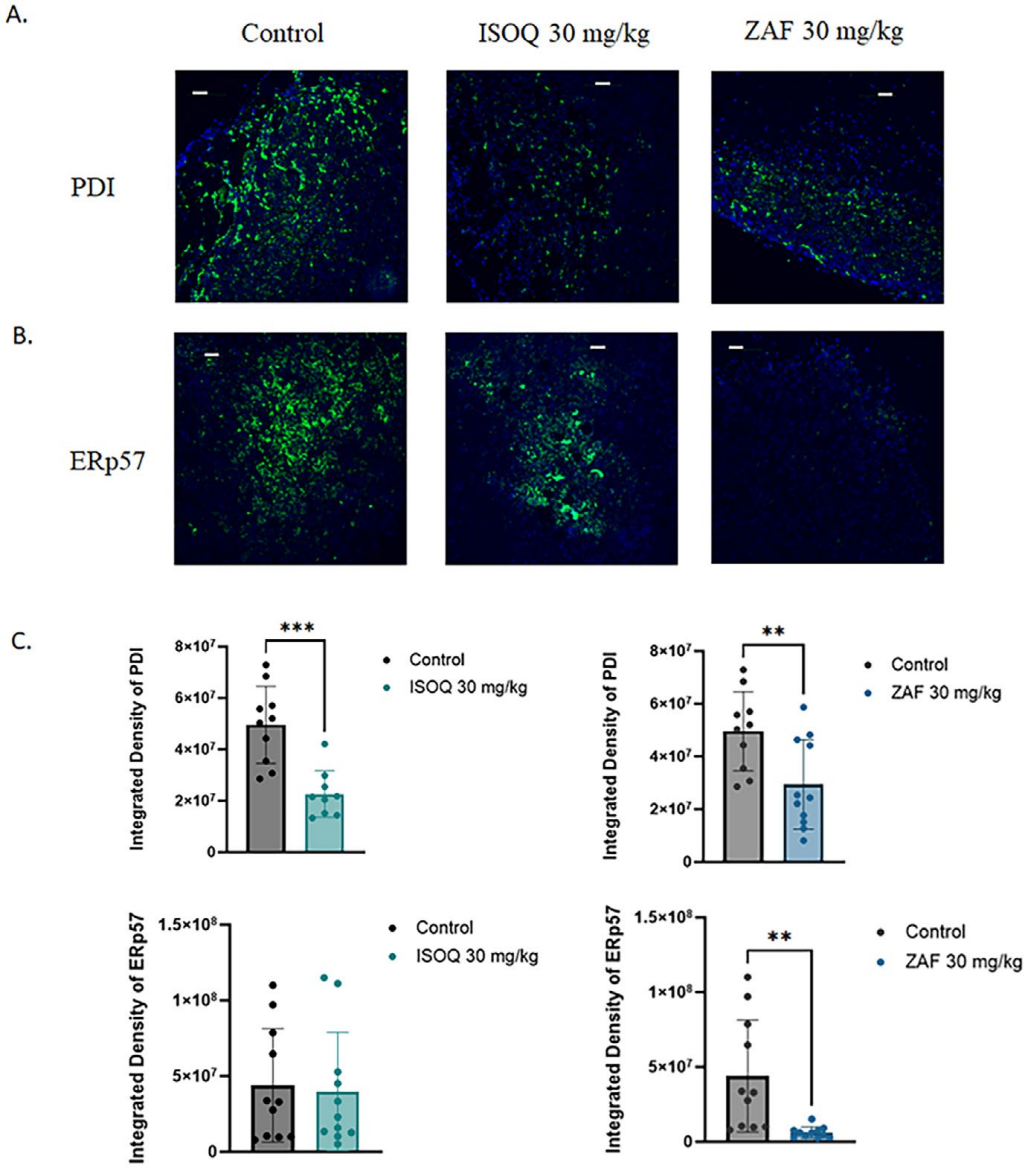
Isoquercetin selectively inhibits PDI, while Zafirlukast preferentially targets ERp57, supporting complementary thiol isomerase inhibition. ISOQ and ZAF's effect on (A) PDI and (B) ERp57 compared to the mock‐treated xenografts. Images are representative of three to five mice per group, with three images taken per mouse of the areas of highest expression. The animals in this experiment are the same as those used in Figures 2 and 3, for ISOQ treatment, and Figure 5 for ZAF treatment. Scale bars are 100 μm. (C) Data are presented as mean ± SD. Statistical analysis was performed using an unpaired *t*‐test, where **p < 0.01** and ***p < 0.001.

Whereas ISOQ is recognized as a selective inhibitor of protein disulfide isomerase (PDI; PDIA1), ZAF has been reported to inhibit a broader set of thiol isomerases, including ERp57 (PDIA3), based on in vitro enzymatic assays using recombinant proteins [12, 14, 37]. To evaluate their relative selectivity in vivo, we performed immunofluorescence analysis of PDI and ERp57 expression in tumors harvested from ISOQ‐treated and ZAF‐treated xenograft models. Consistent with its known target profile, ISOQ significantly reduced tumor PDI levels by 61% (*p* = 0.0002), while having little effect on ERp57 (9% reduction; *p* = 0.8041) (Figure 6). In contrast, ZAF produced a broader inhibition pattern: PDI was reduced by 41% (*p* = 0.0097), and ERp57 was strongly suppressed by 86% (*p* = 0.0034) (Figure 6). These data highlight the complementary enzymatic targets of ISOQ and ZAF, suggesting that their combination may exert enhanced antitumor effects by achieving robust, dual inhibition of both PDI and ERp57.

### 3.6 Isoquercetin and Zafirlukast Potently Inhibit Tumor Growth and Immune Escape Pathways

**FIGURE 7:**
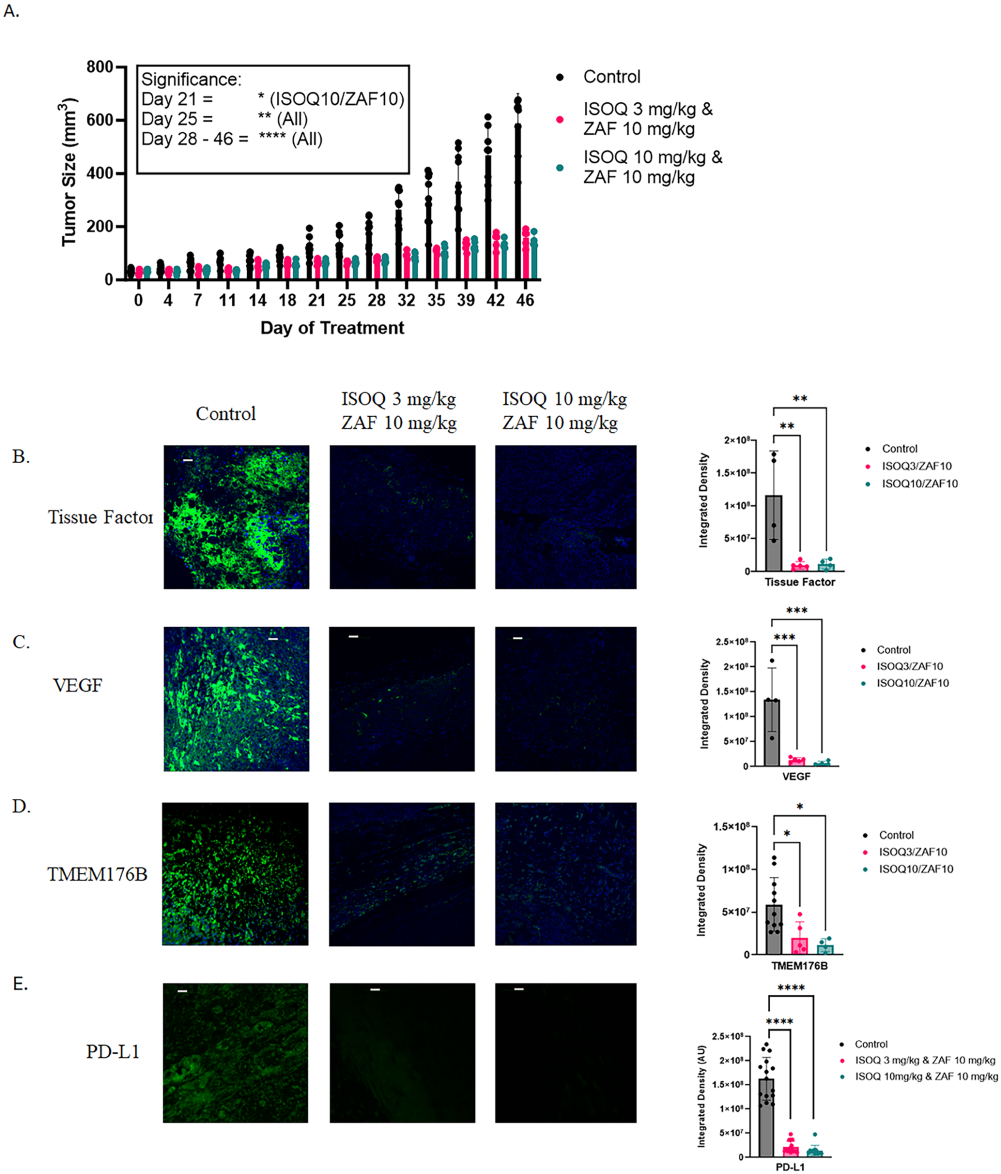
Isoquercetin and Zafirlukast suppress tumor growth and key oncogenic pathways. (A) A combination of 3 mg/kg and 10 mg/kg ZAF (*n* = 8) or 10 mg/kg ISOQ and 10 mg/kg ZAF (*n* = 8) was examined for their effectiveness to inhibit tumor growth in an ovarian cancer xenograft model in NCG mice. Both combinations significantly inhibited tumor growth compared to the control group (*n* = 8). Excised tumors were examined for (B) tissue factor, (C) VEGF, (D) TMEM176B, and (E) PD‐L1 levels of expression. Images are representative of three to five mice per group, with three images taken per mouse of the areas of highest expression. Scale bars are 100 μm. Data are presented as mean ± SD. Statistical analysis was performed using (A) a two‐way ANOVA with Tukey's multiple comparisons test and (B, C, D, and E) using one‐way ANOVA with Dunnett's multiple comparisons test, where *p < 0.05, **p < 0.01, ***p < 0.001, and ****p < 0.0001.

Building on these results, and given their distinct thiol isomerase inhibition profiles, we next evaluated two combination regimens in the OVCAR8 xenograft model using a 3‐fold lower dose of each treatment modality in combination. Thus, since we have used 10 mg/kg and 30 mg/kg of ISOQ and 10 mg/kg of ZAF, the combination groups tested were ISOQ3/ZAF10 or ISOQ10/ZAF10. Both combinations robustly inhibited tumor growth by 73% and 75%, respectively (*p* < 0.0001; Figure 7A). Additionally, immunofluorescence analysis of excised tumors confirmed strong suppression of key tumor‐associated proteins: tissue factor was reduced by 90% in both groups (*p* = 0.003 and *p* = 0.0048 for ISOQ3/ZAF10 and ISOQ10/ZAF10, respectively), VEGF expression was reduced by 90% and 95% (both *p* = 0.0008), TMEM176B by 66% and 80.5% in the ISOQ3/ZAF10 and ISOQ10/ZAF10 groups, respectively (*p* = 0.025 and *p* = 0.0117), and PD‐L1 was reduced by 87% and 91% (*p* < 0.0001 for both groups) (Figure 7B–E).

Compared side by side, the combination regimes consistently outperformed high‐dose (30 mg/kg) ISOQ or ZAF monotherapy across multiple markers (Table 1), drawing on ISOQ monotherapy data (Figure 3), ZAF monotherapy data (Figure 5), and combination results (Figure 7). Compared to high‐dose ISOQ alone, VEGF was reduced by an additional 22% (ISOQ3/ZAF10) and 27% (ISOQ10/ZAF10), TMEM176B by 21% and 36%, tissue factor by 36% in both groups, and PD‐L1 by 5% and 9% respectively (Table 1). Likewise, relative to high‐dose ZAF alone, the addition of ISOQ further suppressed VEGF by 25% (ISOQ3/ZAF10) and 30% (ISOQ10/ZAF10), TMEM176B by 12% and 27%, tissue factor by 16% in both, and PD‐L1 by 3% in the highest group (Table 1). On average, the inhibition of these immuno‐oncology–relevant markers with 30 mg/kg monotherapy with ISOQ or ZAF was ~57%. The combination therapy, using threefold less of each agent, averaged an inhibition of 86.5%, with three of the four markers inhibited by 90% or more (Table 1). These findings highlight a cooperative mechanism of action that amplifies target suppression while potentially reducing toxicity, underscoring the therapeutic potential of dual thiol isomerase inhibition.

**TABLE 1:** Percentage of inhibition for tissue factor, VEGF, TMEM176B, and PD‐L1 for the combination therapies of ISOQ/ZAF compared to either drug alone. The percentage decrease from the control in the immunofluorescence is shown for each of the treatment groups. The data from this table are taken from Figure 3 (ISOQ), Figure 5 (ZAF), and Figure 7 (combination).

	ISOQ 30 mg/kg	ZAF 30 mg/kg	ISOQ 3 mg/kg + ZAF 10 mg/kg	ISOQ 10 mg/kg + ZAF 10 mg/kg
Tissue Factor	54	74	90	90
VEGF	68	65	90	95
TMEM176B	45	54	66	81
PD‐L1	82	88	87	91

### 3.7 Isoquercetin Potentiates Standard Chemotherapy In Vivo

Finally, we assessed whether ISOQ could enhance the efficacy of conventional cisplatin/gemcitabine chemotherapy. We had previously explored the ability of ZAF to do the same, and for this experiment, decided to replicate the conditions we used at that time for a more direct comparison. Thus, we switched the mouse model to NOG mice bearing OVCAR8 xenograft tumors (Figure S1A). ISOQ monotherapy (3 or 30 mg/kg) reduced tumor growth by 49.5% and 58.2%, respectively (*p* < 0.0001). Cisplatin/gemcitabine alone achieved a 71.3% reduction, while its combination with ISOQ further suppressed tumor growth to 77.3% (3 mg/kg ISOQ) and 84% (30 mg/kg ISOQ) (*p* < 0.0001; Figure S1B). Notably, the high‐dose ISOQ combination resulted in tumors 44.3% smaller than chemotherapy alone (*p* = 0.0376; Figure S1B), suggesting that ISOQ enhances the anti‐tumor effect of the chemotherapy. These findings were consistent with our previous data finding that ZAF also enhanced the anti‐tumor effect of the chemotherapy, suggesting that both ISOQ and ZAF could enhance the therapeutic effect of chemotherapy.

## 4 Discussion

This study establishes dual thiol isomerase inhibition as a rational and effective strategy to simultaneously attenuate tumor growth and CAT. Building on the central role of PDI and its family member ERp57 in both coagulation and oncogenic signaling, we show that ISOQ and ZAF, two mechanistically distinct inhibitors of thiol isomerases, exert potent cooperative effects in an ovarian cancer xenograft model.

Our results suggest that selective inhibition of PDI with ISOQ significantly reduces tumor burden and thromboinflammatory markers, while also downregulating tissue factor, VEGF, and other mediators of tumor progression. ZAF, a broader‐spectrum inhibitor of thiol isomerases, including ERp57, similarly reduces tumor growth and key oncogenic markers. When used in combination, ISOQ and ZAF achieved cooperative enzymatic inhibition in vitro and elicited superior antitumor efficacy in vivo, reducing tumor volume and suppressing tissue factor, VEGF, TMEM176B, and PD‐L1 more effectively than high‐dose monotherapy. Importantly, these effects were achieved using one‐third of the monotherapy doses, which would reduce off‐target effects, highlighting the therapeutic potential of dual inhibition. Together, these findings reinforce the concept that thiol isomerases are not merely accessory enzymes but integral components of the pro‐tumor and pro‐thrombotic circuitry in cancer. By targeting both PDI and ERp57, ISOQ and ZAF may disrupt key signaling nodes that tumors exploit to grow, invade, and evade stress [38, 39, 40]. It is likely that this approach not only mitigates the hypercoagulable state common in advanced malignancies but also interrupts thrombosis‐driven remodeling processes that fuel tumor progression. As such, dual thiol isomerase inhibition offers a promising therapeutic strategy to simultaneously target the interdependent processes of cancer‐associated thrombosis and tumor growth.

Our findings further illuminate an intriguing intersection between coagulation, tumor angiogenesis, and immune evasion in the tumor microenvironment. ISOQ/ZAF treatment led to a marked downregulation of VEGF and PD‐L1 in treated tumors. This coordinated suppression is mechanistically significant: VEGF not only drives pathological angiogenesis but also potently suppresses antitumor immunity by impairing dendritic cell maturation, expanding regulatory T cells and myeloid‐derived suppressor cells, and exhausting effector T cells [41]. High VEGF levels in the tumor milieu are a known barrier to immunotherapy [42, 43, 44], and the ability of ISOQ/ZAF treatment to reduce VEGF suggests a more normalized vasculature and improved immune access to the tumor. Simultaneously, lowering tumor and host PD‐L1 levels can relieve T‐cell inhibition and promote immune‐mediated tumor clearance [45, 46, 47]. It is noteworthy that cancer‐associated coagulation can directly contribute to PD‐L1‐mediated immune escape. For example, tissue factor‐factor VIIa signaling through protease‐activated receptor 2 (PAR2) in tumors was recently shown to upregulate PD‐L1 expression and shield breast cancer cells from CD8+ T cell attack [48]. In our study, ISOQ/ZAF treatment significantly attenuated tissue factor activity and expression, which may in turn disrupt this coagulation‐induced PD‐L1 pathway. We also observed decreased expression of TMEM176B in treated tumors, a finding that bridges our therapeutic mechanism to innate immunity. TMEM176B has emerged as a modulator of inflammasome behavior, and when reduced, it can boost cytotoxic T‐cell infiltration and efficacy of checkpoint blockade [35]. By downregulating TMEM176B, the ISOQ/ZAF combination may promote a more inflamed, immunostimulatory microenvironment, thereby priming tumors for improved response to immunotherapies. This mechanistic crosstalk among tissue factor, VEGF, PD‐L1, and TMEM176B highlights how our dual‐targeted approach can recalibrate multiple pro‐cancer circuits at once, blunting the coagulation and angiogenic factors that feed tumor growth and metastasis, while concurrently lifting immune checkpoints that tumors exploit for survival.

In addition to suppressing TMEM176B and PD‐L1, ISOQ markedly reduced expression of key angiogenic and pro‐inflammatory mediators in tumor lysates. Among the most significantly downregulated proteins were matrix metalloproteinases (MMP‐1, MMP‐9) and uPAR, all of which are central to ECM degradation, tumor cell migration, and vascular invasion [49, 50]. Notably, these effects were coupled with an increase in TIMP‐2, suggesting a shift toward a less invasive tumor phenotype [51, 52]. Parallel suppression of inflammatory cytokines and receptors, including IL‐6, IL‐8, sTNF‐RI, and PDGF‐BB, highlights ISOQ's capacity to dampen the pro‐tumorigenic inflammatory milieu. These factors have been linked to pro‐tumor effects, including cancer cell survival, proliferation, myeloid recruitment, angiogenesis, and immune evasion [53, 54, 55, 56], and many are regulated downstream of redox‐sensitive transcriptional pathways linked to PDI and ER stress (e.g., via the PERK/NF‐κB and IRE1/TRAF2 axes) [57, 58, 59]. The coordinated downregulation of these proteins further supports the role of thiol isomerases as upstream orchestrators of tumor‐promoting inflammation and matrix remodeling. Together with our findings on TMEM176B and PD‐L1, these data suggest that thiol isomerase inhibition can reprogram the tumor microenvironment by disrupting both immune evasion and stromal activation, creating a milieu more permissive to immune clearance and less conducive to metastatic spread.

A key strength of this therapeutic strategy is its broad applicability across malignancies. Although our in vivo studies focused on ovarian cancer, the pathways targeted—aberrant thrombosis, angiogenesis, and immune suppression—are hallmarks of many advanced cancers. CAT is a concern, affecting up to 20% of patients with advanced solid tumors within months of therapy [60, 61]. Whereas prophylactic anticoagulation is often underutilized in oncology due to the unacceptable risk of major bleeding with current agents [62], ISOQ/ZAF combination treatment holds considerable translational appeal. Both agents have established safety profiles: ISOQ has shown no increase in bleeding even at high doses that significantly reduced biomarkers of hypercoagulability in cancer patients [31], and ZAF was well tolerated in our Phase I/II trial in patients with ovarian cancer, and no severe adverse events were observed [29]. Notably, in our combination studies, these therapeutic effects were achieved using one‐third the monotherapy dose of each agent, suggesting a favorable safety margin that may further mitigate bleeding risk. This contrasts with VEGF‐targeting therapies—such as bevacizumab and VEGFR‐TKIs like sunitinib—which have been associated with dose‐dependent increases in both all‐grade and high‐grade hemorrhagic events due to their disruption of endothelial integrity [63, 64, 65]. By safely preventing platelet‐driven thrombin generation and fibrin clots, ISOQ/ZAF could fill an important gap in cancer care as an antithrombotic regimen that does not predispose patients to hemorrhage. This benefit may extend beyond reducing VTE incidence by impeding microthrombi and cancer cell–platelet interactions, as well as limiting metastatic seeding and growth at secondary sites.

Equally important is the potential of ISOQ/ZAF therapy to complement or even enhance existing cancer treatments. Given the documented interplay between coagulation, angiogenesis, and immune suppression [66], incorporating ISOQ/ZAF with immune checkpoint inhibitors represents a promising therapeutic strategy. By suppressing VEGF, PD‐L1, and blocking TMEM176B‐mediated immune resistance, ISOQ/ZAF may convert immunologically “cold” tumors into “hot” ones more susceptible to T‐cell attack [67]. A logical next step will be testing ISOQ/ZAF with modern immunotherapies in refractory solid tumors, where synergistic benefits might be realized through this multi‐pronged modulation of the tumor microenvironment. Additionally, our data suggest that ISOQ/ZAF could be deployed as a maintenance or first‐line adjunct therapy to standard chemotherapy. In preclinical models, adding either ISOQ or ZAF to platinum‐based chemotherapy significantly improved tumor growth control (Figure S1 [29]), raising the possibility that patients could achieve deeper or more durable responses with ISOQ/ZAF combination added to standard chemotherapies. For patients who cannot tolerate aggressive chemotherapy, an oral ISOQ/ZAF regimen might even serve as a safer alternative to stabilize the disease while concurrently protecting against thromboembolic events.

In summary, the ISOQ and ZAF combination exemplifies a translational strategy that tackles cancer from multiple angles, including tumor growth, metastasis, thrombosis, and immune escape, using agents with proven safety. Our study provides proof‐of‐concept that targeting thiol isomerases can disrupt the cycle linking cancer and coagulation, potentially improving outcomes across a spectrum of malignancies, not just ovarian cancer. The broader implication is that by intervening at this nexus of hemostasis and tumor biology, we can synergize anti‐cancer therapy with anti‐thrombotic prophylaxis in a way that is both safe and mechanistically comprehensive. As we move forward, ongoing clinical trials and future studies will be critical to determine how this combination can be optimally integrated into cancer care. The encouraging results to date warrant continued investigation of ISOQ/ZAF in larger disease‐specific trials, including those in pancreatic and brain cancers, where thrombosis risk is exceptionally high [68, 69], and in combination with immune checkpoint blockade. Ultimately, this line of therapy could inaugurate a new paradigm of treating the cancer patient as a whole, simultaneously controlling tumor progression and its paraneoplastic complications, to meaningfully improve quality of life and survival.

## Author Contributions

Justine A. Keovilay and Daniel R. Kennedy drafted the manuscript. Justine A. Keovilay performed all experiments. Jason W. Hoskins edited the manuscript. Justine A. Keovilay, Jason W. Hoskins, Thomas C. Lines, and Daniel R. Kennedy designed experiments and edited the manuscript. Daniel R. Kennedy and Thomas C. Lines supervised the project and provided supporting information.

## Funding

This work was supported by a National Cancer Institute grant R21CA231000 to Daniel R. Kennedy. Justine A. Keovilay was supported by a PhD studentship from Quercis Pharma AG. The content is solely the responsibility of the authors and does not necessarily represent the official views of the National Institutes of Health.

## Conflicts of Interest

Justine A. Keovilay and Daniel R. Kennedy receive research support from Quercis Pharma AG. Daniel R. Kennedy is the inventor of a patent on zafirlukast owned by Western New England University and licensed to Quercis Pharma AG. Thomas C. Lines is CEO of Quercis Pharma AG.

## Supporting information


**Figure S1:** Isoquercetin enhances the antitumor efficacy of standard chemotherapy. (A) High and low doses of ISOQ alone and in combination with a chemotherapy regimen were examined in vivo for their effectiveness in an ovarian cancer xenograft model in NOG mice (*n* = 8 per group). (B) At the end of the study, all treated groups significantly inhibited tumor growth compared to the control. (C) A combination of 30 mg/kg of ISOQ and chemotherapy (cisplatin and gemcitabine) significantly inhibits tumor growth, better than chemotherapy alone. Data are presented as mean ± SD. Statistical analysis was performed using two‐way ANOVA with Tukey's multiple comparisons test (A), one‐way ANOVA with Dunnett's multiple comparisons test (B), or an unpaired *t*‐test (C), where *p < 0.05, **p < 0.01, ***p < 0.001, and ****p < 0.0001.

## Data Availability

Privacy/ethical restrictions.
