# Supplementary figures and images for: Isoquercetin and Zafirlukast Cooperatively Suppress Tumor Growth and Thromboinflammatory Signaling in a Xenograft Model of Ovarian Cancer
Source: FASEB J. 2025 Dec 31;40(1):e71361. doi: 10.1096/fj.202502774R (PMC12755188; doi:10.1096/fj.202502774R)

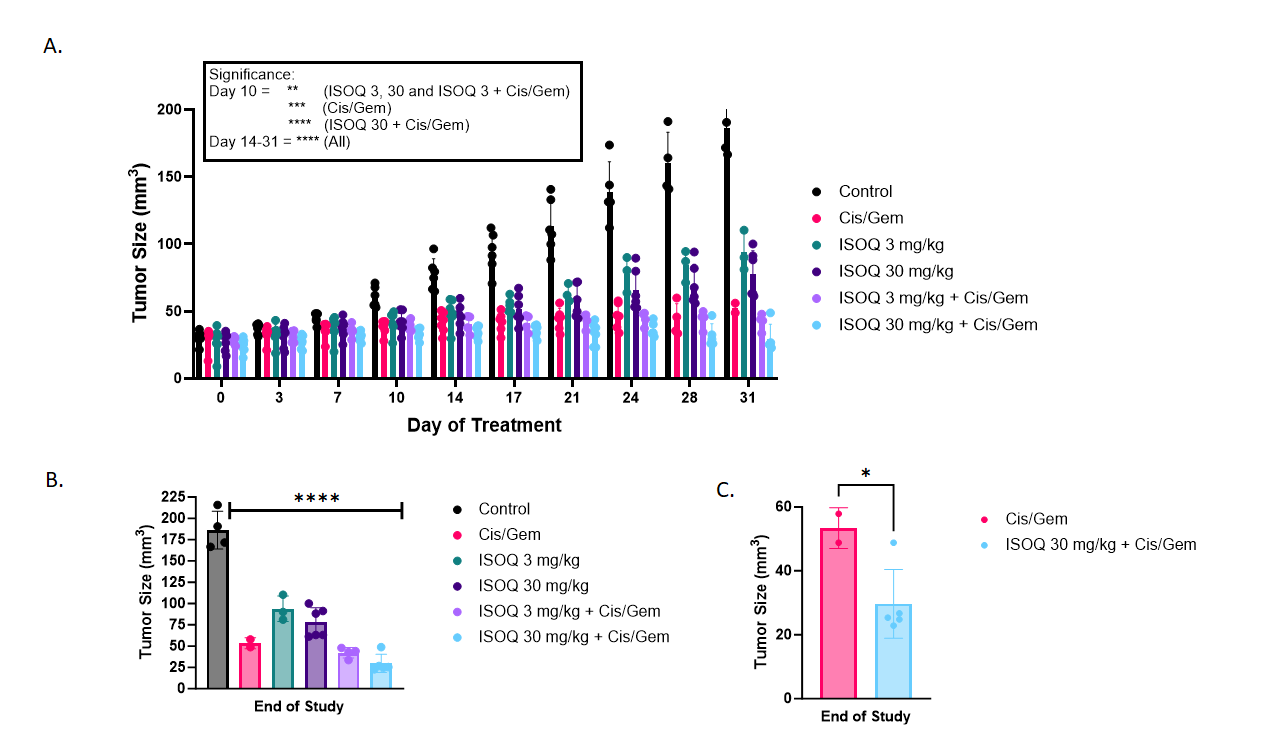

Supplement: Supplementary file 1 — Figure S1: Isoquercetin enhances the antitumor efficacy of standard chemotherapy. (A) High and low doses of ISOQ alone and in combination with a chemotherapy regimen were examined in vivo for their effectiveness in an ovarian cancer xenograft model in NOG mice (n = 8 per group). (B) At the end of the study, all treated groups significantly inhibited tumor growth compared to the control. (C) A combination of 30 mg/kg of ISOQ and chemotherapy (cisplatin and gemcitabine) significantly inhibits tumor growth, better than chemotherapy alone. Data are presented as mean ± SD. Statistical analysis was performed using two‐way ANOVA with Tukey's multiple comparisons test (A), one‐way ANOVA with Dunnett's multiple comparisons test (B), or an unpaired t‐test (C), where *p < 0.05, **p < 0.01, ***p < 0.001, and ****p < 0.0001. [file FSB2-40-e71361-s001.tif]
